# Nano-Catechin Gel as a Sustained Release Antimicrobial Agent against Clinically Isolated *Porphyromonas gingivalis* for Promising Treatment of Periodontal Diseases

**DOI:** 10.3390/biomedicines11071932

**Published:** 2023-07-07

**Authors:** Anahita Javadkhani, Behnaz Shokouhi, Amin Mosayebzadeh, Samira Safa, Mahsa Fahimi, Simin Sharifi, Solmaz Maleki Dizaj, Sara Salatin

**Affiliations:** 1 Dental and Periodontal Research Center, Tabriz University of Medical Sciences, Tabriz 51548-53431, Iransharifi.ghazi@gmail.com (S.S.); 2 Department of Dental Biomaterials, Faculty of Dentistry, Tabriz University of Medical Sciences, Tabriz 51548-53431, Iran; 3 Neurosciences Research Center, Tabriz University of Medical Sciences, Tabriz 51548-53431, Iran

**Keywords:** catechin, nano-catechin, antimicrobial effects, clinically isolated, *Porphyromonas gingivalis*

## Abstract

During the last two decades, new drug delivery strategies have been invented that have been able to solve microbial resistance against antibiotics. The goal of the current report was to assess the antimicrobial effects of nano-catechin gels against clinically isolated *Porphyromonas gingivalis,* one of the main causes of periodontal disease. Catechin-loaded chitosan nanoparticles were prepared by adding a catechin solution to a chitosan solution. Then, the mean particle size and the mean surface charge (zeta potential) of the nanoparticles were detected through photon correlation spectroscopy and zeta sizer, respectively. Nano-catechin gels (1000, 500, 250, 125, 62.5, and 31.2 µg/mL) were prepared, and the antimicrobial assay was performed against clinically isolated *Porphyromonas gingivalis* (*P. gingivalis*). The clinically obtained *P. gingivalis* isolates were obtained from periodontitis patients (N = 15). The consequences are specified as descriptive indices. The normality of data was detected by the Shapiro–Wilk test. Then, to compare the data between groups (with a *p* value < 0.05 as the significance level), SPSS software (version 22) was used via a Mann–Whitney U test. The results showed a nanometer particle size range and a positive zeta potential for the prepared nanoparticles. All the concentrations (1000, 500, 250, 125, 62.5, and 31.2 µg/mL) of nano-catechin gels showed sustained release patterns and were non-toxic against dental pulp stem cells as well. There were no significant differences between the minimal inhibitory concentrations (MICs) for nano-catechin gel (test group) and Chlorhexidine (control group) against 15 isolates (*p* > 0.05). Then, two groups showed similar antimicrobial effects. The similar antimicrobial activity of catechin nanoparticles and Chlorhexidine, as a potent antimicrobial agents, against clinically isolated *P. gingivalis* showed that catechin nanoparticles can be used as a potent antimicrobial material for the treatment of periodontal diseases in the near future.

## 1. Introduction

Periodontitis is one of the most common chronic inflammatory diseases that is triggered by anaerobic bacteria and causes the destruction of the supporting tissue of the teeth. The cause of periodontitis is the presence of anaerobic bacteria that naturally exist in the mouth. Bacteria in the mouth alone cannot cause inflammation, and changes in the host’s immune system response contribute to the development of periodontitis [1]. In periodontal treatments, our goal is to reduce the inflammation as much as possible, restore the gingiva to a healthy state, and restore the gingival tissue using methods to control microbial plaque and other etiological factors. In addition, treatments that modulate the host’s immune system have also had positive effects in this phase of treatment [2].

There are some bacterial strains among the pathogens for periodontal disease, including Capnocytophaga species, *Aggrigatibacter actinomycetemcomitans*, *Porphyromonas gingivalis* (*P. gingivalis*), *Tannerella forsythia*, *Prevotella intermedia*, *Treponema denticola*, Actinomyces species, and *Fusobacterium nucleatum*. Indeed, *P. gingivalis* has always been one of the main reasons for periodontal disease. Some main antimicrobial agents that can be used for effective evading or the treatment of dental and periodontal infections include fluoride (F), calcium (Ca^2+^), as well as phosphate (PO_4_^3−^) and silver (Ag^+^) ions [3].

Nowadays, antibacterial rinsing solutions are frequently used in the treatment of this disease. However, because their durability and duration of action are short, their continuous consumption is required [4]. On the other hand, the increase in microbial resistance against antibiotics available in the market has become one of the main concerns in the field of public health. The concept of bacterial resistance to antibiotics means that microbes become resistant to these drugs through various mechanisms, including gene mutation, and new generations are born where the drug does not have the ability to fight against the same microbe [5]. This phenomenon has finally caused an increase in the need for stronger antibiotics, which can impose very high and back-breaking costs on the pharmaceutical industry and patients. Therefore, it is important to develop controlled-release drug delivery systems with high permeability and longer durability that can deliver antimicrobial and anti-inflammatory substances into the periodontal pocket for a long time [6,7].

During the last two decades, new drug delivery strategies have been invented that have been able to solve these problems to some extent. Due to the high desire for non-invasive drug delivery, direct drug delivery has converted to a current means to administer antibiotics to treat microbial infections [8,9]. Local drug delivery in the target area has many advantages compared to other ways, such as the possibility of using a high concentration of the drug on the tissue, reducing the systemic use of the drug and then reducing the side effects; the possibility of a long-term presence of the drug on the surface and reducing the frequency of use, especially for drugs with a short half-life; easy and painless use; and increased patient acceptance [10,11]. Despite the advantages mentioned above for local drug delivery, only small and hydrophobic molecules can cross the tissue through passive diffusion [7]. Therefore, various strategies have been studied to improve the tissue penetration of therapeutic agents.

**Figure 1:**
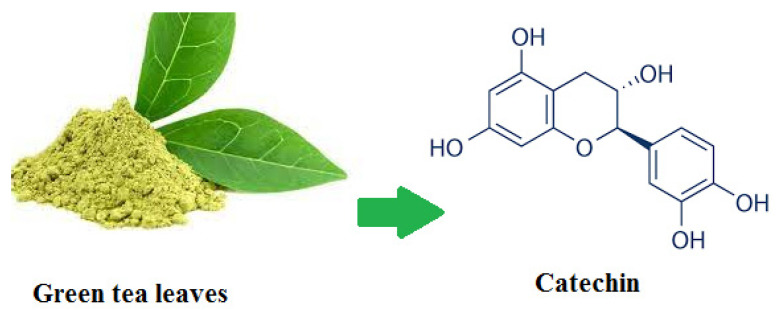
Catechin structure.

Catechin (Figure 1) is the active substance of the green tea plant, which is from the family of flavonoids and is known for its very useful and prominent biological functions such as antimicrobial, anti-cancer, antioxidant, and anti-inflammatory activity. Green tea contains flavonoids, 70% of which are catechins [12]. Toxicity evaluations confirm that catechins are very safe and have no toxic effects, even at high concentrations [13,14].

In addition to strong antimicrobial effects, catechin also reduces gingival inflammation and prevents it from bleeding. Tahani et al. reported in a systematic review that green tea has potential protective effects on oral and dental health due to its catechin and fluoride content. The review and study of eight related articles presented that green tea shows an optimistic result on oral health by dropping the occurrence of caries and periodontal disease due to the presence of catechins [15]. Additionally, in another study, Hirasawa and his colleagues reported significant antimicrobial effects for the hydroxypropyl cellulose drug delivery system containing catechin against *P. gingivalis* bacteria [16]. In another study, Tamura and his colleagues reported that by combining catechin with gel (catechin gel), the antimicrobial action of catechin is extended. They also reported that catechin gel showed inhibitory effects on *Candida* strains as well as *Actinomyces* and *periodontopathic* bacteria [17].

The goal of this report was to assess the antimicrobial effects of nano-catechin gel against clinically isolated *Porphyromonas gingivalis* as one of the main causes of periodontal disease.

## 2. Materials and Methods

### 2.1. Materials

Catechin powder was prepared from Shizuoka, Japan. Chitosan and Tris (2-carboxyethyl) phosphine hydrochloride were purchased from Sigma-Aldrich, Saint Louis, MO, USA. Carbomer 940 was prepared from Millipore Sigma, Germany.

### 2.2. Methods

#### 2.2.1. Catechin-Loaded Chitosan Nanoparticles

One g of chitosan was dissolved in 1000 mL acetate buffer (pH 4.8) to form a 0.1% (*m*/*v*) chitosan solution. Then, using a 5 μm filter, the chitosan solution was filtered. In a separate vessel, 1 g catechin was dissolved in 1000 mL of ethanol. Catechin-loaded chitosan nanoparticles were prepared by adding catechin solution to chitosan solution and stirring (1000 rpm) at room temperature and a pH of 3. After the solutions were added, catechin-loaded chitosan nanoparticles were precipitated by centrifugation at 25,000× *g* for 20 min and freeze-dried (Kimiya Pajuhesh Nanofarnam, Tabriz, Iran) at −80 °C.

#### 2.2.2. Nanoparticle Characterization

The mean particle size and the mean surface charge (zeta potential) of nanoparticles were determined through photon correlation spectroscopy (PCS) and zeta sizer, respectively (Malvern instruments, Herrenberg, UK). A freshly prepared nanoparticles suspension was used for the size and surface amount determination. The fresh suspension of the nanoparticles was ready in distilled water and dispensed into the instrument. For PCS device settings, the laser beam was set at 633 nm and the scattering angle at 90° and 25 °C. PCS is a tool for calculating the hydrodynamic size of molecules, submicrons, and nanoparticles. This examination was performed three times.

One of the important factors for drug-loaded nanoparticles is drug loading percent, which can be defined as the mass ratio of drugs to drug-loaded nanoparticles. For determining the amount of catechin loaded in chitosan, the prepared nanoparticles (10 mg) were sonicated in 100 mL of NaCl solution (0.4 N) for 30 min. A reducing agent (13 mM Tris (2-carboxyethyl) phosphine hydrochloride) was used to prevent degradation of the catechins before assay. Catechin was then assayed using a validated HPLC/UV technique. The main analytical factors of linearity, the limit of detection (LOD), the limit of quantitation (LOQ), and precision were inspected to assess the method’s validation.

#### 2.2.3. Nano-Catechin Gel Preparation

Catechin-loaded chitosan nanoparticles were suspended in distilled water (1% *w*/*w*). Then, 2% *w*/*w* carbomer 940 (Millipore Sigma, Darmstadt, Germany) was added to the suspension and mixed mildly until the gel formed. The gels were prepared for all serial dilutions of the nanoparticles (with concentrations of 1000, 500, 250, 125, 62.5, and 31.2 µg/mL). For determining the concentration of catechin in gels, these concentrations should be multiplied by loading percentage.

#### 2.2.4. The Release Outline

Drug release is the process by which drug solutes travel from their early location in the carrier scheme to the carrier’s outer surface and formerly to the release medium [18]. To govern the outline of drug release from catechin-loaded chitosan nanogels, 300 mL phosphate buffer was transferred into 3 beakers. An amount of 10 mg of the prepared nanogels was poured into each beaker. The pH of the fluid was 7.4, the temperature was 37 °C, and the stirrer was spinning at 100 rpm. Actually, these factors have to be established based on the body’s condition for a dissolution test of a drug (pH of 7.4, temperature of 37 °C, and stirring rate of 100 rpm) [19]. Samples were taken from the beaker every day (one milliliter), and the catechin was then assayed using a validated HPLC/UV technique for 70 days. To maintain the concentration balance, the sample taken from the beakers was replaced with one milliliter of a new buffer medium. Formerly, the cumulative release percentage was calculated against the time (day) for each point. The calculation method for the percentage of cumulative release (%) was according to the below equation:

Cumulative percentage release (%) = Volume of sample withdrawn (mL)/The volume of release media (v) × P (t − 1) + Pt

where Pt is percentage release at time.

#### 2.2.5. Cytotoxicity Test

The cytotoxicity test (cell viability) is aimed to assess the general toxicity of medical devices and materials. This test includes extracting the material in a cell culture media and then exposing the extracted fluid to the cells. Samples were extracted in accordance with ISO 10993-12. The ISO 10993-5, 2009, was used to assess the cell viability. The dental pulp stem cells were used as a test model to evaluate the cytotoxic behavior of nanoparticles. The cells were purchased from the cell bank of Shahid Beheshti University of Medical Sciences, Tehran, Iran. First, the prepared nanoparticles were deposited as disks at the bottom of the wells. The cells were then cultured in DMEM supplemented with serum and antibiotics to obtain a single layer. After incubation for up to 72 h, MTT solution (2 mg/mL PBS) was added to each well and incubated for 4 h at 37 °C. The supernatant was then removed from the wells, and DMSO (200 mL) and Sorenson glycine buffer (25 mL) were added. The absorbance was determined spectrophotometrically at 540 nm and then the cell viability was calculated. The cells without any treatment were used as a control group. Gel itself (without catechin) and catechin powder were tested in terms of cell viability as well.

#### 2.2.6. Sampling of *P. gingivalis*

Here, we clinically isolated *P. gingivalis* from chronic periodontitis patients (N = 15), referring to the Department of Periodontics, Faculty of Dentistry, Tabriz University of Medical Sciences, Tabriz, Iran. After teeth cleaning, the gingival crevicular fluid of the patient was collected from the affected tooth using a sterile filter paper and transferred into a thioglycollate broth medium. All the samples were delivered to a microbiology laboratory as soon as possible after collection and stored at −20 °C until further processing. All patients had written an informed consent form for the collection of *P. gingivitis*.

#### 2.2.7. Cultivation of *P. gingivalis*

The samples isolated from the patients were vigorously vortexed for 30 s. A Columbia agar base supplemented with 5% sheep blood, hemin, vitamin K1, bacitracin, colistin sulfate, and nalidixic acid was used as a selective medium for *P. gingivalis*. Plates were incubated under anaerobic conditions (80% N_2_, 10% CO_2_, 10% H_2_, and 0% O_2_) established by an Anoxomat system (MART microbiology B.V., Drachten, The Netherlands). At 48, 72, and 96 h post incubation time, the growth of bacterial colonies was determined. The presence of *P. gingivalis* on the plates was confirmed using colony morphology and trypsin-like peptidase activity tests.

#### 2.2.8. The Antimicrobial Activity of Nanoparticles

Minimal inhibitory concentrations (MICs) can be defined as the lowest concentration of an antimicrobial agent that inhibits the growth of a microorganism. It can be measured by preparing a dilution series of the antimicrobial agent, adding agar or broth, and then inoculating with bacteria at an appropriate temperature.

In this study, the MICs of the nanoparticles against *P. gingivalis* were examined in the presence of serial dilutions of the nanoparticles (with concentrations of 1000, 500, 250, 125, 62.5, and 31.2 µg/mL) and also Brucella broth enriched with 5 µg/mL hemin, 1 µg/mL vitamin K1, and 5% lysed horse blood. The wells were incubated for 48 h at 35 °C, and microbial growth turbidity was monitored visually. Water and metronidazole antibiotics were used as the negative and positive controls, respectively.

#### 2.2.9. Statistical Analysis

The consequences are specified as descriptive indices. The Shapiro–Wilk test was used to test the normality of the units. Then, SPSS software, version 20 (IBM Company, Armonk, NY, USA), was used to compare the data between groups with a *p* value < 0.05 as the significance level. Mann–Whitney U test was utilized to compare the groups.

## 3. Results

### 3.1. Nanoparticle Characterization

The prepared nanoparticles showed a mean size of 99 ± 1.1 nm (mean for three measurements) and a positive surface charge of +25 ± 2.3 mV (mean for three measurements).

The calibration curve was linear in the ranges between 2 and 40 mg/L, displaying a correlation coefficient (R2) of 0.9989. LOD and LOQ were determined as 4 and 9 standard deviations from the blank signal (N = 7), respectively. The results indicated the method’s validity [18]. Using the calibration data, the loading percentage of catechin in chitosan was obtained: 40.25%.

**Figure 2:**
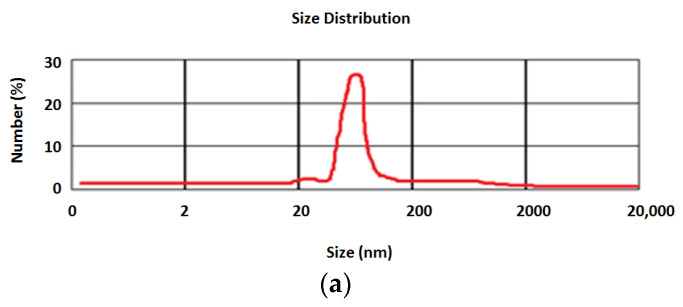

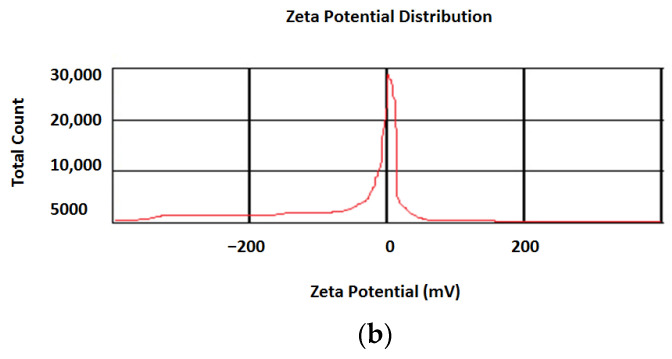
The results for particle size distribution (**a**) and surface charge as zeta potential (**b**).

Figure 2 shows the results for the particle size distribution (Figure 2a) and surface charge as zeta potential (Figure 2b).

### 3.2. Cytotoxicity Test

**Figure 3:**
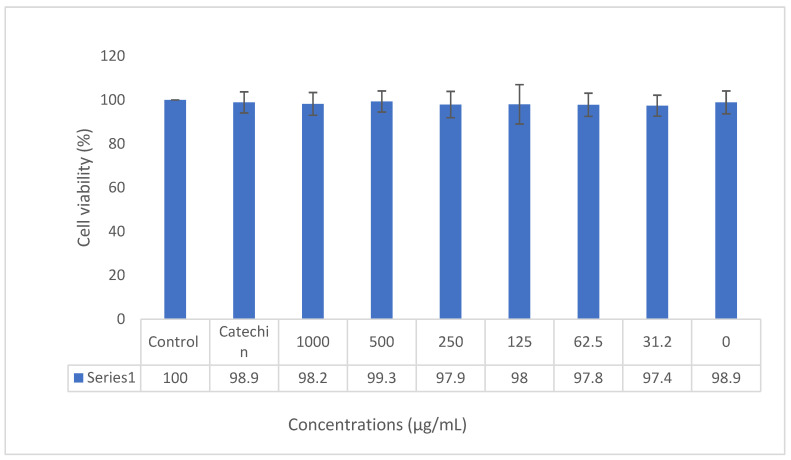
Cell viability results for nano-catechin gels in different concentrations against dental pulp stem cells.

The gels were prepared for all serial dilutions of the nanoparticles (with concentrations of 1000, 500, 250, 125, 62.5, and 31.2 µg/mL). For determining the concentrations of catechin in gels, these concentrations were multiplied by the loading percent (40.25%). Then, the concentrations of catechin in gels were 402.5, 201.25, 100.62, 50.31, 25.12, and 12.55 µg/mL. All concentrations (1000, 500, 250, 125, 62.5, and 31.2 µg/mL) of nano-catechin gels showed non-toxic effects on dental pulp stem cells. Gel without catechin (0%) also had a non-toxic effect on dental pulp stem cells (98.9%). Catechin powder had a viability of 98.9, and the control group (cells without any treatment) had a cell viability of 100% (Figure 3).

### 3.3. Antimicrobial Action

**Figure 4:**
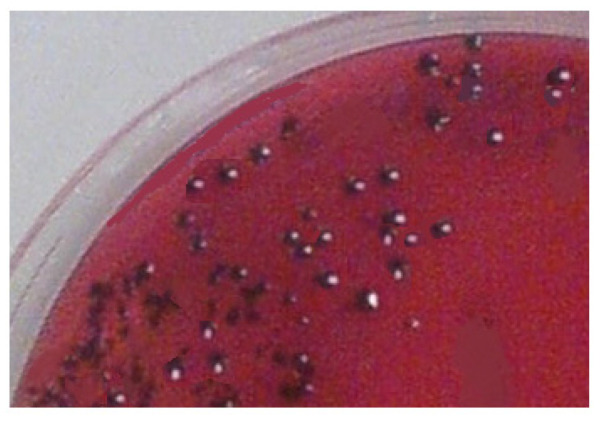
*P. gingivalis* colonies for isolate 1 in our study.

All isolated bacterial colonies of *P. gingivalis* appeared as round, smooth, shiny, and black colonies. Figure 4 shows the *P. gingivalis* colonies for isolate 1 in our study.

**Figure 5:**
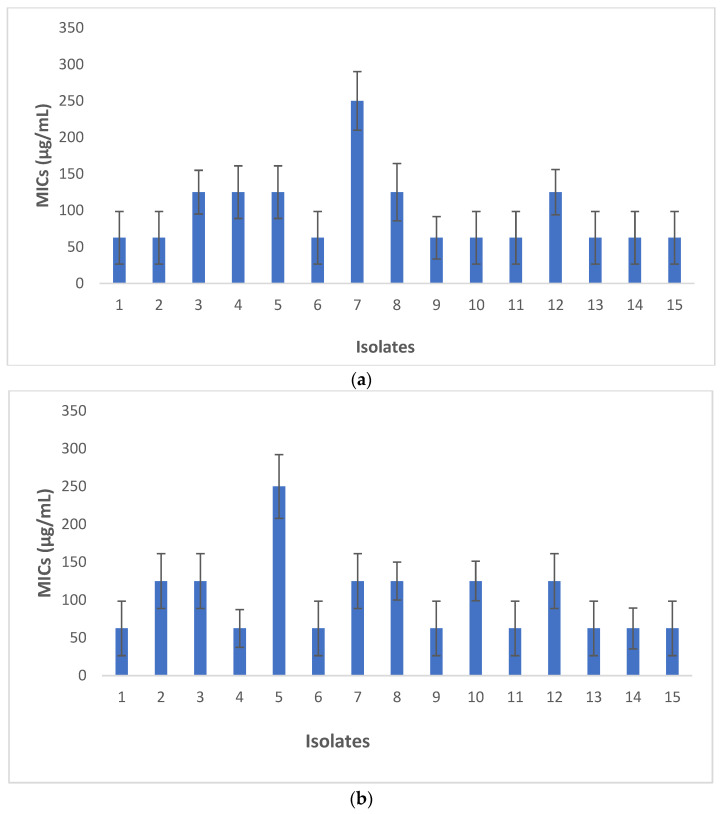
MICs for nano-catechins gel (**a**) and MICs for Chlorhexidine (**b**) for all isolates.

Figure 5a shows the MICs for nano-catechins gel and Figure 5b shows the MICs for Chlorhexidine for all isolates, respectively. There were no significant differences between the MICs for nano-catechins gels and Chlorhexidine against 15 isolates (*p* > 0.05). Then, two groups showed similar antimicrobial effects. The antimicrobial action for gel without catechin against all isolates was zero.

### 3.4. Release Results

**Figure 6:**
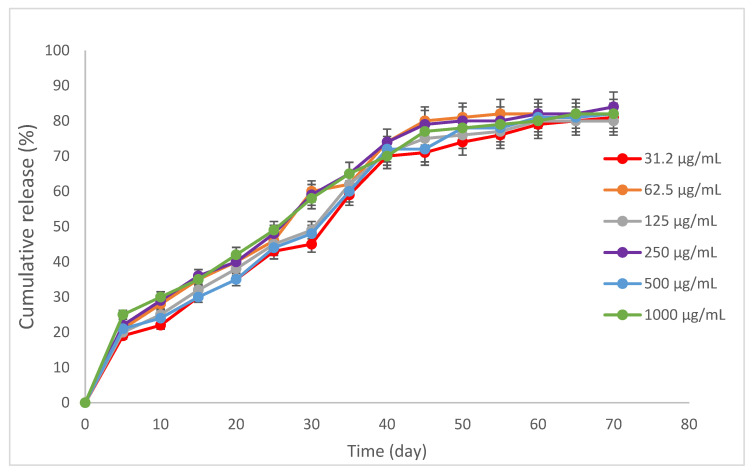
The release pattern for the nano-catechins gel in different concentrations.

All concentrations (1000, 500, 250, 125, 62.5, and 31.2 µg/mL) of nano-catechins gel showed sustained release patterns. Figure 6 shows the release pattern for the nano-catechins gel in different concentrations.

## 4. Discussion

The goal for the treatment of periodontal diseases is to reduce the inflammation as much as possible, restore the gingiva to a healthy state, restore the gingival tissue using approaches to control microbial plaque and other etiological factors. Nowadays, to reduce the possible toxicity and advance the bioavailability of drugs, novel plan-based biomaterials have been developed. Catechin, as an important medicinal plant, has shown good effects in the field of medicine. However, the low bioavailability of catechin is the most important concern for its clinical use. Catechin is also unstable under physiologic conditions [12]. Developing new nano-based formulations for this medicinal plant can help improve its bioavailability and stability.

### 4.1. Nanoparticle Characterization

Assessing the nanoparticles’ characterization is essential for safeguarding their appropriateness for numerous uses, especially in clinical applications. The characterization results for the prepared nanoparticles in this study presented a nanometer particle size range, a positive zeta potential, and a sustained release pattern for the prepared catechin nanoparticles.

The connections and properties of nanoparticles in vitro and in vivo are related to their characteristics [20]. The basic principle is that the properties of materials can dramatically change when a substance’s size is reduced to the nanometer range. Then, the behaviors of passage and the body barriers entering the cell will be changed [20]. The zeta potential can also signify the surface charge in nanoparticles and show their stability behavior. It is often used as a pointer of the particle stability, where an amount of more than ±30 mV specifies high stability against agglomeration. The amounts between 5 and 30 mV show good long-term stability [19]. Then, the prepared particles in this study showed relatively good stability, with a surface charge of +25 mV. In a study by Yue et al., they examined the effect of the surface charge in chitosan nanoparticles on the cellular uptake of eight different cell lines comprising fibroblasts, epithelial cells, endothelial cells, and blood cells. Their results showed that positively charged chitosan nanoparticles (+39.25 mV) presented quicker cellular internalization as well as a greater extent of cellular uptake in all cell lines. The neutrally charged nanoparticles (0.51 mV) and the negatively charged (−45.84 mV) nanoparticles showed low cellular internalization. The authors believed that the obtained results were attributed to electrostatic interactions of positively charged particles with cell membranes [21].

In traditional drug administration, the drug concentration remains within a relatively large range in the blood for a short period of time. This process leads to a low effective dose or exceeds the tolerated dose and drug toxicity. Novel drug delivery systems can maintain the drug dose at the required effective concentration for a long time in the blood through suitable carriers such as nanomaterials. Then, as a result, the frequency of the drug decreases. The drug can show good stability in a carrier, and the drug loaded in the nanocarrier will have a much more noticeable inhibitory effect with long-term drug release compared to the free drug with the same concentration [3]. The determination of drug release rates is influenced by the chemical structure of drugs, which is determined by factors like solubility and molecular weight. These parameters play a crucial role in the diffusion-based delivery and dissolution of drugs from the delivery matrix. Molecules are released from polymeric matrices through mechanisms like Fick’s diffusion, the swelling of the polymer matrix, and the erosion and degradation of the polymer matrix [20].

### 4.2. Cytotoxicity

The cytotoxicity percentage (cell viability) of the produced catechin-containing nanogel on dental pulp stem cells presented that there was no significant decrease in the cell viability of the cells exposed to the nanoparticles compared to the control group. So, the prepared nanogels were non-cytotoxic against dental pulp stem cells. Gel without catechin (0%) also had a non-toxic effect on dental pulp stem cells.

### 4.3. Antimicrobial Effects

There were no significant differences between the MICs for nano-catechins gels and Chlorhexidine against 15 isolates (*p* > 0.05). Then, two groups showed similar antimicrobial effects. For nano-catechin gels, isolate number 7 and, for Chlorhexidine, isolate number 5 showed the highest MICs of 250 µg/mL. The MIC values of catechin in other studies were reported in the range of 62.5–250 μg/mL as well [13,14].

In this regard, Lagha et al. reported the ability of tea catechins to increase the barrier integrity of *P. gingivalis* epithelium and protect against *P. gingivalis*-induced epithelial disruption [22]. Lee et al. demonstrated the anti-inflammatory effects of catechin by reducing IL-1β levels in a periodontitis mouse model induced by *P. gingivalis* [23]. Asahi and co-workers reported that catechin damages the cell membrane of *P. gingivalis* and prevents biofilm formation. According to their MIC results, the catechin effects on *P. gingivalis* biofilms were dose-dependent (*p* < 0.0001) [24]. In another research work, the MIC values demonstrated the preventive and therapeutic effects of catechin to inhibit the growth and adherence of *P. gingivalis* to oral epithelial cells in a dose-dependent manner. Catechins were also able to enhance the potential of antibiotics (metronidazole and tetracycline) and decrease the expression of *P. gingivalis* genes implicated in host colonization [25].

Upon infection of the periodontal tissue by periodontal pathogens, macrophages produce proinflammatory cytokines like IL-1, IL-6, and TNF-α, which play crucial roles in regulating the inflammatory response to combat the infection. However, the excessive production of inflammatory cytokines has been associated with periodontitis [26].

Catechin has been found to effectively eliminate *P. gingivalis* by significantly reducing the production of genomic DNA, protein, carbohydrates, and RNA in the extracellular polymeric substances (EPS) of bacterial biofilms [27]. Additionally, catechin can decrease the expression of three genes (hagA, hagB, and fimA) in *P. gingivalis* that code for virulence factors involved in colonizing the host [25]. Furthermore, catechin exhibits anti-inflammatory effects by suppressing the production of proinflammatory cytokines IL-1β and TNF-α induced upon *P. gingivalis* infection [23]. It also irreversibly damages the bacterial cell membrane and effectively prevents *P. gingivalis’* adherence to oral epithelial cells, thereby reducing colonization at subgingival sites [25]. Moreover, it inhibits the upregulation of matrix metalloproteinase expression in osteoblasts induced by *P. gingivalis* extracts and hinders the enzymatic activities of *P. gingivalis* [28].

Catechin’s suppressive effect on IL-1β and TNF-α production may provide a beneficial approach for modulating periodontitis [23]. Previous studies have reported that catechin suppresses inflammation through the NF-κB-mediated pathway, which is also influenced by the MAPK pathway [24,25,26,27,28,29]. Among various MAPKs, p38 MAPK is particularly important in regulating inflammatory responses, and its activation is crucial for the release of proinflammatory cytokines like IL-1β and TNF-α. Pretreatment with catechin attenuates the activation of NF-κB induced by *P. gingivalis* and specifically inhibits the phosphorylation of the p38 MAPK, among other MAPKs. Catechin treatment also reduces the activation of TLR2 and TLR4 induced by *P. gingivalis*, along with the expression of related adaptor molecules such as MyD88, TRIF, and TRAF6. Furthermore, the significant attenuation of IL-1β production by catechin is observed in TLR2 and/or TLR4 knockout cells, suggesting that downregulating TLR signaling contributes to the anti-inflammatory properties of catechin [22,23]. The activation of the inflammasome, which converts pro-IL-1β to IL-1β, serves as the second signal for IL-1β release. The inflammasome consists of NLR or AIM2 family receptors and procaspase [23].

## 5. The Strengths and Limitations

The outcomes of the current in vitro study showed that nano-catechin gel had appropriate antibacterial activity against *P. gingivalis*. This result might actually be valuable in overcoming bacterial resistance. In total, the MICs found in this study were lower compared to those gained in prior studies, proceeding with the optimism about preparing ideal designs based on these nanoparticles.

The probability of mistakes in the sampling of bacteria, nanoparticle collection, and microbial contamination with other bacterial strains can be mentioned as the main study limitation. There are also other types of bacteria that act as periodontal pathogens. The prepared nanogels in this study should also be tested and advanced against these bacteria in future research. In total, this study was an in vitro report. More investigations should be tested in future studies on animals or as clinical trials. Furthermore, the exact antimicrobial and antibiofilm mechanisms for them should be examined to determine their real function.

## 6. Conclusions

The characterization results presented a nanometer particle size range (99 ± 1.1 nm), a positive zeta potential (+25 mV), a loading percentage of 40.25%, and a sustained release pattern for the prepared catechin nanoparticles. All concentrations (1000, 500, 250, 125, 62.5, and 31.2 µg/mL) of nano-catechin gel showed no significant antimicrobial action against 15 isolates compared to the control group (Chlorhexidine). The similar antimicrobial activity of catechin nanoparticles and Chlorhexidine, as potent antimicrobial agents, against clinically isolated *P. gingivalis* showed that catechin nanoparticles can be used as a potent antimicrobial material for the treatment of periodontal diseases in the near future. More studies are needed in future studies on animals or as clinical trials for this material to be beneficial in periodontitis therapy.

## 7. Future Perspectives

Nano-based dental products have been shown to be more beneficial than conventional materials to show better capabilities in many aspects. The novel antimicrobial materials are brilliant in all arenas of dental action. Owing to their usefulness, they are a helpful resource in clinical dentistry for a variety of uses. There are some studies that have achieved significant results. Some kinds of nano-based products have been directed toward industrial manufacture. But examinations of the real action mechanisms and founding processes of the microstructure of nanomaterials still remain undiscovered, and many parts need to be uncovered. Due to the distinctive properties of nanomaterials, their wide-ranging use predictions and huge potential value in the future of dentistry will be measured. Advancing new formulations of plant-based nano-antimicrobials together with their other beneficial actions like reductions in gingival inflammation and the prevention of its bleeding can be helpful for the periodontitis therapy field. Then, it is required to save the way of evolving new nanomaterials and enlarge our knowledge of their mechanisms to improve their properties.

## Data Availability

The raw data for this study can be shared at this time.
